# Mastoiditis: Causes, Prevention and Treatment

**Published:** 1904-03

**Authors:** J. M. Crawford

**Affiliations:** Atlanta, Ga., Surgeon to Presbyterian Hospital, Tabernacle Infirmary, Halcyon Retreat, and Hospital for Incurables


ATLANTA
Journal-Record of Medicine.
Successor to Atlanta Medical and Surgical Journal, Established 1855,
and Southern Medical Record, Established 1870.

VOL. V. MARCH, 1904. NO. 12.

BERNARD WOLFF, M.D., EDITOR, Nos. 319–20 Prudential.

Published Monthly.

M. B. HUTCHINS, M.D., BUSINESS MANAGER, 1007-1008 Century Bldg.

### ORIGINAL COMMUNICATIONS.

### MASTOIDITIS: CAUSES, PREVENTION AND TREATMENT.*

BY J. M. CRAWFORD, M.D., ATLANTA, GA.,
Surgeon to Presbyterian Hospital, Tabernacle Infirmary, Halcyon Retreat, and Hospital for Incurables.

In discussing this subject, I must necessarily repeat many things that I have said before. It is a subject to which I have paid much attention since 1895, and as I have made fifty-six operations for this trouble I will confine my remarks to my own observations and experience.

The position of the mastoid process is familiar to all. It lies behind the ear and is a cellular bony structure, with a hard bone inner and outer table, varying much in structure in the adult, but its general shape is much the same, being somewhat triangular with the apex downward. It contains a certain amount of diploë, and a number of pneumatic cells besides the antrum. There

*Read before Atlanta Society of Medicine, February 4, 1904.

is a small orifice that leads to the middle ear, in its upper portion, to the antrum or largest cell of the mastoid, known as the aditus. Through this channel the product of imflammation passes from the middle ear to the mastoid, and thereby setting up mastoiditis. Hence, we have this affection secondary to suppuration of the middle ear.

Of the fifty-six operations I have made, by far the greater number of cases were the result of *acute* suppurative aural catarrh, a good portion being secondary to scarlet fever, while the smallest number could be attributed to *chronic* suppuration of the middle ear. No one, young or old, is exempt from this affection where there is a discharging ear.

The beginning of a case of mastoiditis is marked by a cold, the same causing earache. This is the time for the abortive treatment. Inflate the middle ear, and by all means lose no time by the use of drops of whatever character in the external auditory canal. I have found the application of ice to the mastoid to be of little service. To prevent mastoiditis you must first prevent the suppuration in the middle ear, and my experience has been that inflation of the middle ear is the best method to pursue, since it allows the secretion to pass out through the Eustachian tube. For this purpose I seldom use the Eustachian catheter, but use instead compressed air from an air receiver or tank, at a pressure of about thirty-five pounds. I have found no trouble to arise from this method, and prefer it to the catheter, inasmuch as it is much more powerful and more quickly manipulated.

As the Eustachian tube is not only an air-conductor to the middle ear, but is also a drainage-tube, you can readily see why I lay so much stress upon inflation, or the opening of the natural outlet—Eustachian tube—especially when it is known that when this method is employed perforation of the drum membrane is impossible.

If the satisfactory results of inflation in cases of acute aural catarrh were better known, there would be fewer advocates of the old-time prescription of laudanum and sweet oil, a perfectly useless procedure, since it does not reach the affected point. Every general practitioner should equip himself with a Politzer air-bag or use in cases of earache, as by it the Eustachian tube which is closed by engorgement can be readily opened, and relief quickly procured. Should the inflation in the case of threatened mastoiditis prove unsuccessful, incising the drum membrane should be resorted to in cases where perforation has not already occurred, or where a small perforation exists. Bearing in mind that mastoiditis is liable to occur whenever there is a discharging ear, it does seem that the profession would wake up to the fact that such a condition is not a matter of little importance, but that on the contrary it should receive prompt attention until the discharge had ceased in cases where it had already begun, and to be prevented in cases where the perforation had not already been made. Would they not also be a little more careful and not allow patients with a running ear from scarlet fever to mix with other children before this discharge had ceased? At least to prevent such children from mixing with others until we consider there is no danger of a contagion from this running ear. I have seen a few cases where I could not account for the contagion other than this source.

The patient should be kept in bed and free from worry. Watch him carefully. Should swelling of the mastoid arise, or should there occur much pain on pressure over the mastoid, or should a very anxious facial expression be manifested, then I consider an operation is necessary.

The most prominent symptoms met with in mastoiditis, and upon which most dependence can be placed, are local tenderness over the mastoid region and a sagging of the superior posterior wall of the external auditory canal, close to the tympanic ring. We may have them combined, but either one alone often constitutes the only sign upon which the necessity of operative procedure is based. Meningitis is sometimes the result of mastoiditis, and hence a symptom not to be overlooked. The temperature can not always be relied upon as a symptom, since I have made the operation in cases where this was not above normal.

In determining mastoid tenderness, care must be taken to be sure that the pain experienced by the patient is mastoid tenderness and not tenderness from the external auditory canal. Where there is tenderness and swelling, there is one trouble with which mastoiditis may be confounded, namely, furuncle of the external auditory canal. Most usually in cases of furuncle, the swelling is located anteriorly, while in mastoiditis the swelling is posteriorly. A very good point, too, is to press the finger backward and inward upon the mastoid just behind the insertion of the auricle. This procedure does not move or press against the canal. On examination the most tender point is situated over the antrum.

The instruments required in this operation are : A medium-sized scalpel, curved scissors, fixation forceps, retractors, rawhide mallet, various-sized chisels, rongeur forceps with different-sized blades, a small probe, sharp spoon-curettes, periosteum elevators, and artery forceps. The ear should be thorougly cleansed and tamponed with bichloride gauze ; the mastoid region shaved and thoroughly cleansed with bichloride solution, 1 to 1000, and covered with towels dipped in the same solution. All instruments should be sterilized by boiling, and the operator's and assistant's hands receive the necessary attention. Strict antiseptic principles should be observed throughout the operation, for we do not know when we are to enter the brain cavity.

The primary incision begins about half an inch below the tip of the mastoid process and is carried upward close to the line of the insertion of the auricle to a level with the superior extremity of the auricle. The line of incision should be made close to the line of the insertion of the auricle, so that when the flaps are retracted the superior walls of the external auditory canal are freely exposed. The incision made in this way also heals up, leaving very little deformity. Throughout the entire incision, the soft part, including the periosteum, should be divided to the bone. From about the center, and at right angles to the primary incision, a second incision about an inch is made directly backward. This second incision makes two posterior flaps, which renders it very much easier to be elevated and pushed out of the field of operation. The superior posterior is now elevated and pushed upward and backward, well out of the way. The anterior flap is then elevated and pushed forward until the posterior and superior margins of the external auditory canal are clearly seen. The post inferior flap is now elevated and pushed backward and downward. With blunt, curved scissors the sterno-mastoid is next cleared from the tip of the process.

In this procedure the scissors should be made to hug the bone very closely so as not to wound any of the large vessels of the neck.

Hemorrhage in this operation is never very great. I seldom find it necessary to tie a vessel, torsion only being necessary. A retractor is now placed behind the anterior flap and held forward by the assistant. The flap being pushed well out of the way, the wound is sponged out by the assistant and the operator finds his anatomical landmark.

The antrum is best located by bearing in mind its relation to the superior and posterior walls of the external auditory meatus. If a triangle is formed, one line of which is drawn horizontal with the root of the zygoma, another vertical and tangent to the posterior wall of the external auditory canal, then finish triangle with straight line; this triangle is known as post-meatal triangle. This triangle lies immediately over the antrum.

Having located the antrum we proceed to enter it. The cortex is best removed by means of the mallet and chisel; a large curved chisel is used at first, being applied to the surface of the skull, and is made to cut away the bone in thin, broad chips. In this way we form a bony channel, the center and deepest part of which should correspond to the antrum. We know that the antrum is entered by its depth. A probe placed in the artificial opening and then placed in the external auditory canal will find both to be of the same depth. With rongeur forceps and sharp spoon-curette, the whole of the pneumatic structure of the mastoid process is obliterated, and all overhanging bone removed. The operation should be continued until sound bone is encountered in every direction. After all of the cells have been removed and the operation completed, the wound is packed with bichloride gauze. Before this, however, I find it well to place a few stitches in the wound, one stitch being placed at the superior extremity of the incision. The second incision is closed up entirely. I find this to give better results than to leave the wound entirely open. Bichloride gauze is then placed over the wound, on top of which is placed a piece of sterile cotton and held in place with a bandage. In cases where the technique has been perfect it is not necessary to remove the dressing under five or six days. At each dressing, which should be about five days apart, the wound and the external auditory canal should be well irrigated with a hot bichloride solution, 1 to 5,000.

Exposure of the lateral sinus is not a very dangerous occurrence, but when it does occur, it is difficult to proceed with the operation. I have had only six cases where the lateral sinus was accidentally exposed. Fortunately for me in these cases I have just mentioned, the operations were about completed when I undertook to ascertain the size of the opening in the cranial cavity, and I had the misfortune to lascerate the walls of the internal lateral sinus. The bleeding in these cases was immediately checked by a compress made of gauze, which was not removed for seven days, at which time there was no bleeding upon dressing the wound.

There are now in use two methods of operative procedure, one known as the Schwartze, which is performed only in acute cases, and the other known as the radical operation, where it is intended to remove the whole of the diseased bone. In the former, the original incision is really the same as in the radical operation, the only difference is in the extent of the incisions being shorter. The mastoid cells are all removed, leaving the bony wall that lies behind the mastoid cells and the external auditory canal. In the radical operation this bony wall is removed, throwing the middle ear, the external meatus, the attic and antrum into one large cavity.

Permit me to say just here that prior to 1895 the only operative method that was used in Atlanta, so far as I know, for this affection was what is known as Wild's incision, a semi-circular cut behind the ear, which extended through the skin and at places probably through the periosteum. After this was done a rude dressing was applied, and the surgeon waited the consequences. If the patient survived it was all right; if not, the only thing that could be said was that the doctor had done all that was in his power to do. You can now see the folly of such a position.

Generally, no difficulty is experienced in making the diagnosis; on the other hand, we meet with cases in which we are in doubt. I was called to a case of this character some time ago by Dr. Huguly, while he was at Barnesville, Ga. The patient had no signs whatever of mastoiditis, but marked signs of meningitis and a running ear. Absolutely, there was no swelling over the mastoid, nor pain on pressure. The history of the case was this: About two weeks before, Dr. Huguly was called to see the patient, who was suffering with great pain in the ear. After continuing to suffer from such pain for several hours, the drum membrane ruptured and the ear began to run. Even at this time there was marked tenderness over the mastoid which lasted for about three days, when the swelling and pain gradually subsided. Marked symptoms of meningitis, however, followed this recession of pain and swelling. For this reason only I decided to operate. On entering the antrum I found a great amount of pus, but no opening into the brain cavity. This patient is now well; I feel confident that she would have died soon had not the operation been made.

Case 2: H. E. R., age thirty, presented himself January 16, 1902, with acute suppurative aural catarrh of both ears, with pain behind both ears. He gave a history of having an operation made on right mastoid two years before. I treated him daily until March 6th, when I decided to operate on him for mastoiditis. There was but little pain on pressure and no swelling over mastoid. I made the operation and found the inner table to be necrosed, really a good-sized hole into the cranial cavity. This patient would not have survived had not the operation been made.

Case 3: On May 14, 1900, Mrs. E. M. S. came to my office presenting marked symptoms of acute aural catarrh of the right ear. Hoping to avert an operation I sprayed the nose and throat and inflated the tympanic cavity. She returned the next morning no better; at which time I incised the drum membrane well and found pus. When she came in the next morning I soon saw that an operation upon the mastoid would be needful. I made an arrangement to operate the next day. Upon my arrival I found that the symptoms, both pain and swelling, had disappeared. She stated to me that about one o'clock the night before, relief came suddenly. I could get no tenderness over the mastoid, nor did she present any other symptoms of mastoiditis. However, I operated and found a great amount of pus, also an opening into the brain cavity, the rupture of which was accountable for her relief. This patient would have died had she not had the operation made. I am in favor of early and radical operative procedure.

Case 4: M. C., age seven and one-half, came to my office last July with a running left ear, three years' duration, from scarlet fever. I put her on antiseptic treatment for this affection and had her to carry it out for about six months. On finding no cessation of this discharge, advised radical operation left mastoid, which I made under ether, finding a large mastoid antrum with pyogenic membrane. In this case the aditus was unusually large, but not not large enough to prevent of free drainage. Here the operation was made to remove the danger from a mastoiditis.

My plan will be hereafter in cases that I can control to treat cases of running ears for not longer than six months, and if not cured, to make the radical operation.

So you will readily see that some of our most dangerous cases are those that present the least number of symptoms. This is explained by the position of the necrosis. The nearer the inner table the more dangerous, while at the same time we see fewer symptoms of mastoiditis.

